# Association Between Vitamin D and Resistin in Postmenopausal Females With Altered Bone Health

**DOI:** 10.3389/fendo.2020.615440

**Published:** 2021-01-15

**Authors:** Sundus Tariq, Saba Tariq, Saba Khaliq, Mukhtiar Baig, Manal Abdulaziz Murad, Khalid Parvez Lone

**Affiliations:** ^1^ Department of Physiology, University Medical & Dental College, The University of Faisalabad, Faisalabad, Pakistan; ^2^ Department of Physiology and Cell Biology, University of Health Sciences, Lahore, Pakistan; ^3^ Department of Pharmacology and Therapeutics, University of Health Sciences, Lahore, Pakistan; ^4^ Pharmacology and Therapeutics, University Medical & Dental College, The University of Faisalabad, Faisalabad, Pakistan; ^5^ Clinical Biochemistry, Faculty of Medicine, Rabigh, King Abdulaziz University, Jeddah, Saudi Arabia; ^6^ Department of Family Medicine, Faculty of Medicine, King Abdulaziz University, Jeddah, Saudi Arabia; ^7^ Department Physiology and Cell Biology/Metabolic Disorders, University of Health Sciences, Lahore, Pakistan

**Keywords:** resistin, vitamin D, osteoporosis, bone health, postmenopausal women

## Abstract

**Background:**

Resistin is a relatively novel adipokine that has a role in bone remodeling and may regulate bone mineral density (BMD). Vitamin D and adipokines have a dynamic role in the body’s various metabolic processes, including bone metabolism, and may alter bone metabolism in relation to each other. This study aimed to investigate the association between vitamin D and serum resistin levels in postmenopausal non-osteoporotic and osteoporotic females.

**Methods:**

This correlational analytical study was conducted on 161 postmenopausal females, divided into two groups, non-osteoporotic and osteoporotic, between 50–70 years. Bone mineral density (BMD) was assessed by dual-energy X-ray absorptiometry (DXA) scan. Serum resistin and vitamin D levels were analyzed by enzyme-linked immunosorbent assay (ELISA) method. Serum calcium, phosphate, and alkaline phosphatase with spectrophotometry. A correlation was checked using spearman’s rho correlation coefficient, and multivariate stepwise regression analysis was used to predict serum resistin levels.

**Results:**

Postmenopausal females (n=161) having sufficient, insufficient and deficient levels of vitamin D were 87 (54.0%), 64 (39.8%), and 10 (6.2%), respectively. Lumbar spine BMD (p < 0.001), total hip BMD (p < 0.001), and serum resistin levels (p < 0.001) were significantly different between the two groups. There was a significant negative correlation between serum resistin and vitamin D in postmenopausal females (rho = -0.182, p = 0.021) and osteoporotic group (rho = -0.253, p = 0.019) but non-significant in non-osteoporotic group (rho = -0.077, p = 0.509). Serum vitamin D was found to be independent predictor of serum resistin levels, accounting for only 3% variance.

**Conclusion:**

Serum vitamin D levels were low while serum resistin levels were high in postmenopausal osteoporotic females and vitamin D is a negative predictor of serum resistin levels.

## Introduction

Osteoporosis is one of the major epidemics of the 21^st^ century and has affected more than 200 million women globally (1). It is a skeletal disease affecting bone strength, quality and increases fracture risk. Primary osteoporosis that occurs in the postmenopausal group is referred to as a metabolic disorder of bone, caused by a disparity and contrast between the functions of osteoclast (bone-resorbing cells) and osteoblast (bone-forming cells) (2). It is a multifactorial disease and has been linked to vitamin D deficiency and changes in serum levels of various adipokines. Optimal vitamin D levels, calcium, and other nutrients are essential to maintain proper bone health (3).

Vitamin D deficiency is quite prevalent, especially in postmenopausal women, and worldwide almost one billion people are suffering from vitamin D insufficiency or deficiency (4). The inability to activate vitamin D with advancing age might cause insufficient levels in postmenopausal women (5).

Vitamin D is stored and metabolized in adipose tissue (6), releasing special chemical messengers called adipokines. These adipokines like leptin, resistin, and adipsin, regulate the body’s metabolic processes, including bone metabolism. The adipokine, resistin, is not only expressed in the adipocytes but many other cells of the body as well, including monocytes and bone marrow cells (7). Serum resistin is believed to negatively affect bone metabolism by increasing osteoclastogenic activity and is determined to be the independent predictor of low bone mineral density (BMD) (8). The increase in osteoclastic activity by high serum resistin levels is attributed to its proinflammatory role by increasing the release of several proinflammatory cytokines (9). At the same time, vitamin D is well known for its role in reducing or regulating proinflammatory cytokines (10). Thus, there may be an association between vitamin D and serum resistin levels that may further lead to osteoporosis development or progression, especially in postmenopausal females. This study has been designed to evaluate vitamin D’s association with serum resistin levels in postmenopausal non-osteoporotic and osteoporotic females.

Osteoporosis is a major cause of low trauma fracture that can lead to poor life quality, loss of height, self-esteem, kyphosis, significant morbidity, mortality and burden on society. Thus, early identification of the contributing factors and taking proper measures to control them is important to reduce the burden of this disease.

## Materials and Methods

This correlational analytical study was conducted for two years (2018–2020), after taking ethical approval from the Institutional Review Board of the University of Health Sciences, Lahore, Pakistan. Postmenopausal women (n=161) with at least 2 years of amenorrhea and age between 50 to 70 years were recruited from outpatient department of Madina Teaching Hospital, Faisalabad, and divided into two groups. Postmenopausal non-osteoporotic women (n=75) having T-score ≥ -1.0 and postmenopausal osteoporotic women (n=86) with T-score ≤ -2.5. Both groups were age, BMI, and gender-matched. The matching performed was not blinded. Written informed consent was obtained from all study participants. General and demographic information of study subjects were taken by qualified personnel. Height in meters (m) and weight in kilograms (kg) were measured using standard procedure, and BMI was calculated by dividing weight in kgs over height in m^2^. Women with chronic renal or liver disease, malignancies, autoimmune disease, iatrogenic or premature menopause, and medications affecting bone mineralization and taking vitamin D supplements were excluded from the study.

### Bone Mineral Density

BMD of postmenopausal females was evaluated at the Pakistan Institute of Nuclear Medicine (PINUM) Hospital, Faisalabad, Pakistan by using HOLOGIC-HORIZON-A (QDR-series) version 5.6.0.4, dual-energy X-ray absorptiometry (DXA) system. The areal BMD is estimated by DXA in g/cm^2^ that quantifies the skeletal status.

According to the criteria set by the World Health Organization (WHO), osteoporosis in adults is diagnosed by the T-scores obtained from DXA. T-score is defined as comparing measured BMD results with the average BMD of the young adults at the time of peak bone mass. T-score ≤ 2.5 standard deviations below the mean peak bone mass were considered as osteoporosis. T-scores included in the study were quantified from the lumbar spine (L1 to L4) and total hip.

### Biochemical Parameters

Five ml blood was withdrawn from selected subjects, centrifuged for 10 min at 3,000 revolutions per minute to obtain serum. We ran the tests to detect the biochemical parameters at the biochemical laboratory of Madina Teaching Hospital and the postgraduate research laboratory of The University of Faisalabad, Pakistan.

Serum calcium, phosphate, and alkaline phosphatase were measured by the colorimetric method, using Roche diagnostics, Cobas 6000, COBI-CD, manufactured by Hitachi High Technologies Corporation, Tokyo, Japan. Serum vitamin D and resistin levels were quantified by human 25(OH) vitamin D and human resistin enzyme-linked immunosorbent assay (ELISA), formulated by PerkinElmer and Elabscience Biotechnology Inc., respectively. The assay was performed using microplate data collection and analysis software Gen5™ and Gen5 Secure, manufactured by BioTek^®^ Instruments, Inc. The sensitivity, coefficient of variation, and cross-reactivity of serum resistin assay were 18.75 pg/ml, <10%, and almost nil, respectively, as reported by the manufacturer.

The sensitivity, intra, and inter-assay coefficient of 25(OH) vitamin D assay variation was reported to be 0.397 ng/ml and <100%, respectively by the manufacturer. The cross-activity of 25(OH) vitamin D assay to both 25(OH) vitamin D2 and 25(OH) vitamin D3 was 100% as reported by the manufacturer. Vitamin D levels were classified into three, depending on the information provided in the kit literature. Subjects having vitamin D levels < 10 ng/ml, 10–30 ng/ml, and > 30 ng/ml were classified as deficient, insufficient, and sufficient, respectively.

### Statistical Analysis

Data were entered and analyzed in statistical package for social sciences (SPSS) version 22. The normality of the data was checked by the Kolmogorov–Smirnov test. Non-parametric quantitative data was represented in the median (IQR). Mann-Whitney U test was applied for comparison between non-osteoporotic and osteoporotic groups. A Chi-square test was applied for comparison between proportions. Spearman’s rho correlation coefficient was used to evaluate correlations between various variables and 2-tailed significance was taken. Multivariate stepwise regression analysis was used to predict serum resistin levels. A p-value of ≤ 0.05 is considered statistically significant.

## Results

The median age of non-osteoporotic and osteoporotic postmenopausal females was 57 yr (50-64) and 57 yr (52.75–65), respectively that was statistically non-significant. The anthropometric measures, lumbar spine BMD, total hip BMD and biochemical parameters of both groups are given in **Table 1**. Only lumbar spine BMD (p < 0.001), total hip BMD (p < 0.001) and serum resistin levels (p < 0.001) were significantly different between the two groups.

**Table 1:** General, anthropometric, and biochemical parameters of study groups.

Parameters	Non-osteoporotic (n=75)	Osteoporotic (n=86)	p-value
Age (years)	57 (50–64)	57 (52.75–65)	0.063
Menopausal age (years)	50 (49–54)	50 (48–52)	0.112
Height (m)	1.55 (1.50–1.57)	1.52 (1.50–1.57)	0.891
Weight (kg)	66 (55–74)	67.50 (60.00–75.25)	0.260
BMI	27.42 (22.91–31.66)	27.96 (25.18–31.75)	0.242
Lumbar spine BMD	0.20 (-0.50 to 1.00)	-2.80 (-3.30 to -2.50)	0.000*
Total hip BMD	0.50 (-0.25 to 1.00)	-1.85 (-2.35 to -1.43)	0.000*
Serum Resistin (ng/ml)	2.01 (0.64–4.33)	6.51 (1.86–10.25)	0.000*
Serum Vitamin D (ng/ml)	32.16 (19.17–52.01)	30.83 (21.06–45.52)	0.293
Serum Calcium (mg/dL)	9.50 (9.30–9.90)	9.60 (9.27–9.90)	0.953
Serum Phosphate (mg/dL)	3.80 (3.40–4.20)	3.90 (3.40–4.20)	0.644
Serum Alkaline Phosphatase (U/L)	97.00 (79.00–120.00)	96.00 (82.50–119.25)	0.895

Values are given as median (IQR).

*p-value ≤ 0.05 is considered statistically significant.

**Figure 1:**
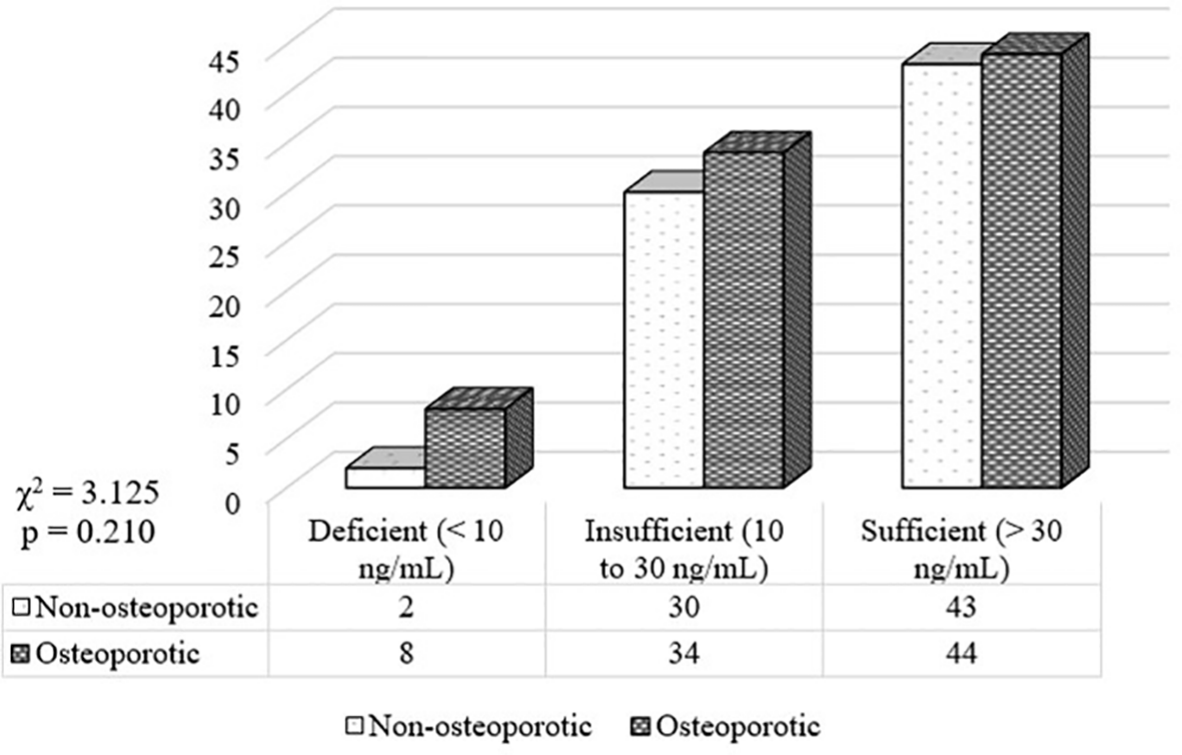
Frequency distribution of vitamin D among the study groups.

Postmenopausal females (n=161) having sufficient, insufficient and deficient levels of vitamin D were 87 (54.0%), 64 (39.8%), and 10 (6.2%), respectively. Postmenopausal non-osteoporotic females (n=75) having sufficient, insufficient and deficient levels of vitamin D were 43 (57.3%), 30 (40.0%), and 2 (2.7%), respectively. Postmenopausal osteoporotic females (n=86) having sufficient, insufficient and deficient levels of vitamin D were 44 (51.2%), 34 (39.5%), and 8 (9.3%), respectively (**Figure 1**).

Correlation of serum resistin levels with anthropometric measures, biochemical parameters and BMD in postmenopausal non-osteoporotic and osteoporotic females was checked using spearman’s rho correlation coefficient (**Table 2**).

**Table 2:** Correlation of serum resistin levels with anthropometric measures, biochemical parameters, and BMD in postmenopausal non-osteoporotic and osteoporotic females using spearman’s rho correlation coefficient.

Parameters	Non-osteoporotic		Osteoporotic	
	rho	p-value	rho	p-value
Age	0.279	0.015*	-0.003	0.978
Menopausal age	0.197	0.090	-0.013	0.906
Height	-0.190	0.102	0.147	0.177
Weight	-0.055	0.641	0.043	0.696
BMI	-0.030	0.797	0.024	0.829
Vitamin D	-0.077	0.509	-0.253	0.019*
Calcium	-0.081	0.487	0.021	0.847
Phosphate	0.097	0.407	-0.004	0.972
Alkaline phosphatase	-0.008	0.948	-0.196	0.070
Lumbar spine BMD	0.085	0.469	-0.039	0.720
Total Hip BMD	-0.001	0.994	-0.050	0.650

Correlation is seen using spearman’s rho correlation coefficient.

*p-value ≤ 0.05 is considered statistically significant (2-tailed).

**Figure 2:**
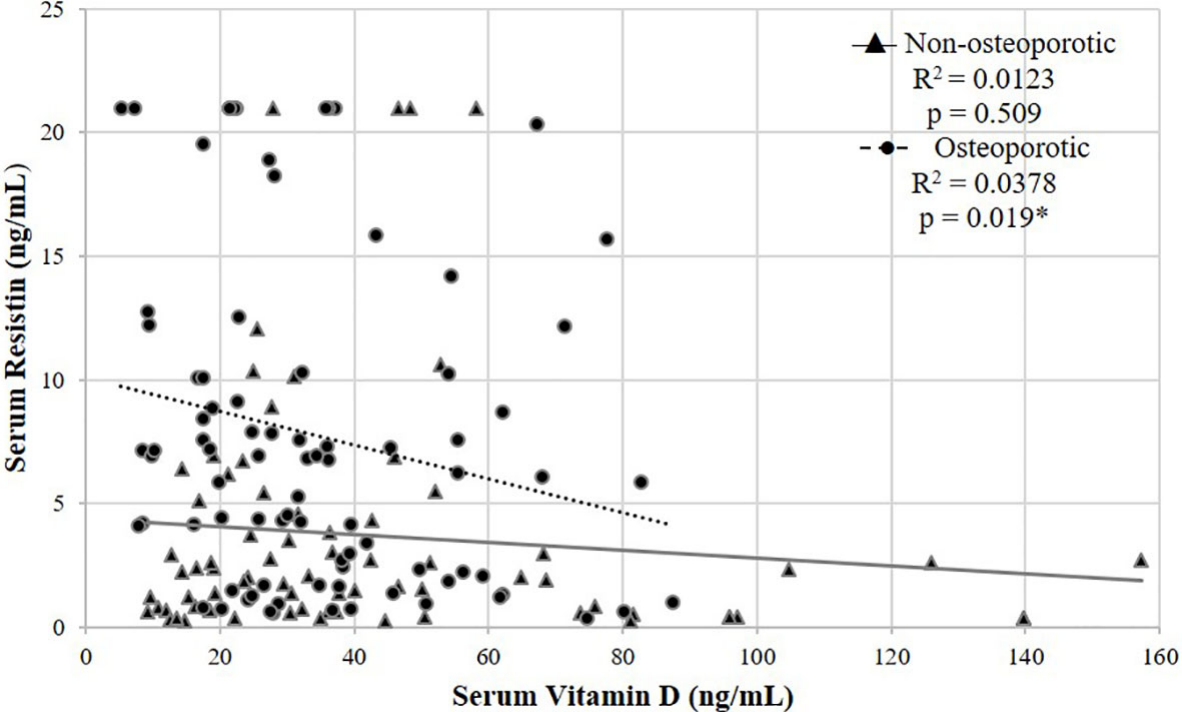
Scatter plot showing a negative correlation between serum resistin and vitamin D in postmenopausal non-osteoporotic and osteoporotic females using spearman’s rho correlation coefficient. *p < 0.05 is statistically significant.

The correlation between serum resistin and vitamin D was non-significant in postmenopausal non-osteoporotic females (rho = -0.077, p = 0.509), while this correlation was statistically significant (2-tailed) in postmenopausal osteoporotic females (rho = -0.253, p = 0.019) (**Table 2**, **Figure 2**), which remained significant after adjusting for age, menopausal age, height, weight and BMI (rho = -0.215, p = 0.05).

**Figure 3:**
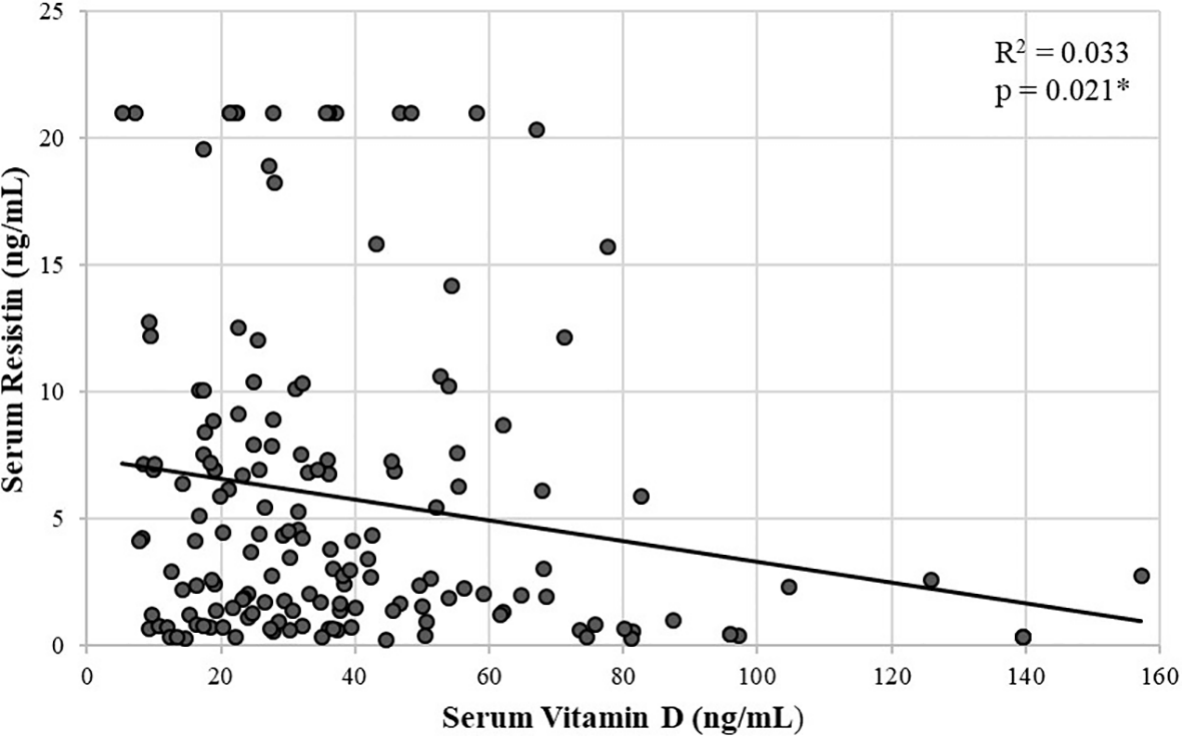
Scatter plot showing a significant negative correlation between serum resistin and vitamin D in postmenopausal females using spearman’s rho correlation coefficient. *p < 0.05 is statistically significant.

There was also significant negative correlation between serum resistin and vitamin D in postmenopausal females (rho = -0.182, p = 0.021) (**Figure 3**). The correlation remained significant after adjusting for age, menopausal age, height, weight, and BMI (rho = -0.211, p = 0.008).

Age, menopausal age, height, weight, BMI, serum vitamin D, calcium, phosphate, and alkaline phosphatase were used in a multivariate stepwise regression analysis to predict serum resistin levels. The model accounted for approximately 3% of the variance (R^2^ = 0.034, Adjusted R^2^ = 0.28). The independent predictor was only serum vitamin D levels, accounting for only a 3% variance in serum resistin levels (**Table 3**).

**Table 3:** Multivariate regression analysis showing independent predictors of serum resistin levels in postmenopausal females.

Dependent variable	Model	Predictors	β coefficient	Std. Error	R^2^	sr^2^	*p-*value
Serum Resistin	1	Constant	7.387	0.834	–	–	0.000
		Vitamin D	-0.041	0.017	0.183	0.033	0.020

Excluded variables were age, menopausal age, height, weight, BMI, serum calcium, phosphate, and alkaline phosphatase.

p-value ≤ 0.05 is considered statistically significant.

sr^2^ is the squared semipartial correlation.

## Discussion

Adipokines’ crucial role in different physiological processes, including inflammation, immune function, glucose and lipid metabolism, and bone homeostasis, has been revealed in many studies in recent years. A number of data indicate that adipokines can influence bone remodelling processes and are involved in osteoporosis’s pathogenesis (11).

In the present study, serum vitamin D levels were comparable between the non-osteoporotic and osteoporotic groups. Similarly, few other studies demonstrated no significant difference in mean values of vitamin D between control and osteoporosis postmenopausal women (12, 13). In contrast to our results, several studies have reported significantly lower levels of Vitamin D among osteoporotic postmenopausal women compared to the control group (14, 15). Literature suggested that vitamin D levels depend on many variables, including nutrition, sun exposure, age, eating patterns, lifestyle, lack of screening systems, metabolic, genetic, cultural and social causes, and other environmental variables (13, 16, 17).

With vitamin D deficiency, the risk of osteoporotic fracture is increased (18). In the current study, we found that around half of our postmenopausal females had either insufficient or had deficient levels of vitamin D. Several other studies in Pakistan found that most postmenopausal females were deficient in vitamin D levels (4, 19, 20). In Pakistan, there is plenty of sunlight throughout the year that is a prerequisite for synthesizing vitamin D in the skin. So, our study participants’ deficient and insufficient levels indicate that several other contributing factors are also implicated.

This study also found a significant negative relationship between serum resistin and vitamin D levels in postmenopausal osteoporotic females. Similar to our results, another study reported a negative association of vitamin D with serum resistin (21). However, this association was not observed in a few other studies (22, 23). Therefore, it is apparent that the data is dual in terms of the relationship between vitamin D and resistin. The difference between ours and their study is that the negative correlation between vitamin D and resistin was found in a subgroup of higher BMI patients. Another study found an inverse relationship between resistin levels and vitamin D levels in patients with type 1 DM. However, they did not find any specific mechanism (24).

In contrast to our results, another study found a positive association of vitamin D with serum resistin levels. However, they observed these higher resistin levels with sodium restriction. The higher resistin levels with higher 25(OH)D status might directly be linked to dietary restriction of sodium in their study (25). Our results found that only vitamin D predicts serum resistin levels in postmenopausal osteoporotic females, reinforcing the idea of vitamin D ‘s involvement in regulating resistin. It seems that vitamin D might be implicated by an unexplained mechanism in resistin’s modulation (24). At present, it is difficult to formulate the clinical studies’ relevance of such types of cross-sectional studies. We need further molecular level studies and longitudinal clinical trials to determine the clinical significance of estimation of resistin among osteoporotic subjects.

We also found increasing levels of serum resistin in postmenopausal osteoporotic females. In line with our results, another study found increased resistin levels in menopausal women with osteoporosis than a healthy group (26). The plausible cause for this could be that in osteoporosis, the proinflammatory cytokines such as serum levels of IL-6, leptin, IL-1, and resistin increases and might be the main contributing factor in the pathogenesis of osteoporosis. This observation emphasizes the significance of early intervention in the state of osteoporosis to stop its progression. Research also showed that resistin, leptin, IL-1, IL-4, IL-6, and TGF-β played a significant role in postmenopausal women’s bone metabolism and could be used as a diagnostic marker capable of recognizing patients at risk of osteoporosis and thereby predicting the risk of fracture. Interventions such as anti-cytokine therapy may be used in the future to treat osteoporosis (9). It has been shown that resistin plays a role in bone remodeling by inducing osteoclastogenesis through NF-κB signaling activation, thereby promoting bone resorption processes (27).

Another mechanism through which resistin can affect bone regulation is its effect on osteocalcin, secreted by osteoblast, and is a potential biomarker of bone formation. A study stated that serum resistin has a negative correlation with osteocalcin, and when in osteoporosis levels of serum osteocalcin decreased in return, the levels of resistin increased (28). These results may indicate that resistin controls osteocalcin by negative feedback: resistin suppresses osteocalcin development and is secreted in response to low levels of osteocalcin (28).

There was no significant difference between calcium and alkaline phosphatase in postmenopausal non-osteoporotic and postmenopausal osteoporotic group. Similar to our results, another study found no apparent effect of vitamin D deficiency on BMD and serum calcium, phosphorous, and alkaline phosphatase (20). The influence of resistin on bone and its relationship with vitamin D tends to vary. The mechanisms that underlie these changes are not fully known. It is essential to achieve a more precise explanation between vitamin D and resistin relationship so that new targets can be identified to treat osteoporosis.

## Limitations

Osteoporosis is a multifactorial disease. Therefore, bone markers associated with osteoporosis could have been measured in this study. A cross-sectional design, smaller sample size, inclusion of only female subjects and the use of ELISA in place of mass spectrometry to measure vitamin D levels were other limitations of the study.

## Conclusion

Serum vitamin D levels were low while serum resistin levels were high in postmenopausal osteoporotic females and vitamin D is a negative predictor of serum resistin levels.

## Data Availability Statement

The raw data supporting the conclusions of this article will be made available by the authors, without undue reservation.

## Ethics Statement

The studies involving human participants were reviewed and approved by Ethical review committee of The University of Faisalabad, Pakistan. The patients/participants provided their written informed consent to participate in this study.

## Author Contributions

****SuT: Conception and design, acquisition, analysis, interpretation of data, and drafted the manuscript. ****SaT: Acquisition, analysis of data, carried out the literature search, and helped in drafting the manuscript. SK:**** Designed and supervised the research, and revised the manuscript critically for important intellectual content. MB:**** Carried out the literature search, analyzed the data, and revised the manuscript. MM:**** Contributed to manuscript drafting and data analysis KL:**** Designed and supervised the research, and revised the manuscript critically for important intellectual content. All authors contributed to the article and approved the submitted version.

## Conflict of Interest

The authors declare that the research was conducted in the absence of any commercial or financial relationships that could be construed as a potential conflict of interest.
